# Dispositional Optimism, Burnout and Their Relationship with Self-Reported Health Status among Nurses Working in Long-Term Healthcare Centers

**DOI:** 10.3390/ijerph17144918

**Published:** 2020-07-08

**Authors:** Maria Carmen Malagón-Aguilera, Rosa Suñer-Soler, Anna Bonmatí-Tomas, Cristina Bosch-Farré, Sandra Gelabert-Viella, Aurora Fontova-Almató, Armand Grau-Martín, Dolors Juvinyà-Canal

**Affiliations:** 1 Department of Nursing, University of Girona, Health and Healthcare Research Group, 17003 Girona, Spain; carme.malagon@udg.edu (M.C.M.-A.); anna.bonmati@udg.edu (A.B.-T.); cristina.bosch@udg.edu (C.B.-F.); aurora.fontova@udg.edu (A.F.-A.); dolors.juvinya@udg.edu (D.J.-C.); 2 Department of Nursing, University of Girona, 17003 Girona, Spain; sandra.gelabert@udg.edu; 3 Quality of Life Institute, University of Girona, 17003 Girona, Spain; grauma@comg.cat

**Keywords:** health psychology, health promotion, occupational mental health, optimism, burnout, elderly care, long term care

## Abstract

The mental health of nurses working in long-term healthcare centers is affected by the care they provide to older people with major chronic diseases and comorbidity and this in turn affects the quality of that care. The aim of the study was to investigate dispositional optimism, burnout and self-reported health among nurses working in long-term healthcare centers. A descriptive, cross-sectional survey design was used. Survey questionnaires were distributed in 11 long-term health care centers (*n* = 156) in Catalonia (Spain). The instruments used were LOT-R (dispositional optimism), MBI (burnout) and EuroQol EQ-5D (self-reported health). Bivariate analyses and multivariate linear regression models were used. Self-reported health correlated directly with dispositional optimism and inversely with emotional exhaustion and cynicism. Better perceived health was independently associated with greater dispositional optimism and social support, lower levels of emotional exhaustion level and the absence of burnout. Dispositional optimism in nurses is associated with a greater perception of health and low levels of emotional exhaustion.

## 1. Introduction

Nurses and midwifes compose 71% of the world’s health professionals [1], and their health is of interest to the professionals themselves, health service administrators and political representatives. The role of nurses in residences for the care of patients with severe chronic diseases and mental health issues is to support and help patients in the process of recovering from illness, to guide them in care-related matters and to help them undertake daily life activities [2,3]. Nurses who work in the care of the elderly at long-term healthcare centers have a high level of work satisfaction due to their professional autonomy and their good relations with users, families and professional colleagues [4], Despite this, it has been observed that nurses in elderly care have a sense of loneliness in their daily work for different reasons: firstly, they care for a greater number of patients than nurses working in acute-care settings, which in itself creates a stressful environment; secondly, the number of registered nurses in this setting is lower than in other areas of care and this frequently results in treatments being directly applied by nursing assistants, which leaves registered nurses to act more as managers with few opportunities to exchange impressions with other nurses like them and often requiring them to rely exclusively on their own skills [4]. Other studies have highlighted, through the words of the nurses themselves, the lack of technical resources and the difficulties derived from the physical limitations of patients in geriatric care settings [5]. It is difficult for nurses with a high level of stress to maintain a positive emotional state and to feel fulfilled with their professional activity [6,7].

In workplaces, optimism is being studied as a personality trait that permits workers to remain motivated and that has repercussions on behavior within an organization [8,9,10]. Dispositional optimism, defined as a stable and generalized tendency, expectation or belief that positive things will happen in life, is considered a personal characteristic of interest in nurses [11]. Optimistic people face the problems and demands that occur on a daily basis in a healthier manner and are more resistant to the biological and psychological effects of stress and illness than pessimistic people [12,13,14,15]. A recent study demonstrated the association between greater optimism and lower mortality in nurses [16]. Furthermore, optimistic people have greater psychological resistance and are more capable of recovering from an adverse event with greater efficacy than less optimistic people [17,18]. Dispositional optimism is positively associated with self-reported health [12] and negatively associated with burnout in nurses [19].

In 2019, the World Health Organization included burnout as a professional disease in the International Statistical Classification of Diseases and Related Health Problems (ICD-11) [20]. Burnout in some professionals is considered to be a state that encompasses emotional exhaustion, cynicism and reduced professional efficacy [21], which has negative repercussions on organizations. Specifically, emotional exhaustion is found in those situations in which workers perceive that they are unable to give more of themselves emotionally due to work demands. Cynicism appears when negative attitudes and feelings appear towards the beneficiaries of one’s work. Reduced professional efficacy at work is defined as the tendency of professionals to evaluate themselves negatively, affecting their capacity to perform their work and to relate to the people they serve [22]. Nurses are one of the groups of healthcare workers with the worst perception of health as a result of burnout [7] and it has been established internationally that nurses from different care areas have high percentages of burnout [23,24], associated to the loss of health and wellbeing [25].

In summary, the health of nurses in the workplace is internationally relevant. Nurses are subjected to a large number of occupational stressors [26,27,28]. According to the International Labour Organization [29], this occupational stress is recognized as a factor that negatively affects people’s health. Nurses are known to be a group of workers with a high incidence of burnout [7]. Positive traits such as dispositional optimism influence physical health [16] in coping with stress and consequently affect occupational health [12]. The relationship between these variables has been studied in hospital nurses but has not been addressed in geriatric nurses in Spain [7]. The hypothesis of this study is that nurses in long-term healthcare who have high levels of optimism and self-social support also have better perceived health and less burnout. The aim of the present study was to investigate dispositional optimism and burnout and their relationship with the self-reported health of nurses working in long-term healthcare.

## 2. Methods

### 2.1. Design

This study used a descriptive, cross-sectional survey design between May 2014 and February 2015.

### 2.2. Participants

The Spanish social and health care model includes comprehensive, global, interdisciplinary, universal and equitable care in the services provided and in the resources used. It promotes the principles of autonomy and participation of individuals and families and guarantees continuity of care. This healthcare model makes a comprehensive assessment of the person, family and environment. In this research, we included all nurses working in the long-term healthcare centers in Girona (11 centers, *n*: 156), regardless of the type of their employment contract. Nurses who only performed outpatient care and those professionals who were on temporary sick leave at the time of data collection were excluded from the study.

### 2.3. Measures and Instruments 

The self-report questionnaire consisted of three instruments and demographic, occupational and health-related variables (age, sex, socioeconomic level, civil status, with dependent family members, chronic health problems, self-perceived social support, employment status and conditions, receiving continuous training and work-related family conflicts).

Dispositional optimism (LOT-R) [30] is the revised questionnaire by Scheier et al. [31] consisting of 10 items with five Likert-type responses. Scores range from 5 (in complete agreement) to 1 (in complete disagreement). Only 6 items are scored (1, 3, 4, 7, 9 and 10), three are phrased positively (1, 4 and 10; in the direction of optimism) and three are phrased negatively (3, 7 and 9; inverse direction of optimism). The other 4 items (2, 5, 6 and 8) are distracters that are not scored. Scores between 6 and 30 points can be obtained. High scores in the LOT-R are considered to imply optimism [30]. The internal validity and consistency of the Spanish version of the questionnaire is 0.72 for the whole of the questionnaire [32].

MBI-Maslach questionnaire [33]. The Maslach and Jackson questionnaire [34] consists of 22 items spread over three subscales or dimensions (emotional exhaustion, cynicism and professional efficacy) with six Likert-type options, from “Never” to “Every day”. The emotional exhaustion dimension has 9 items and a maximum score of 54. The cynicism dimension has 5 items and a maximum score of 30. The professional efficacy dimension has 8 items and a maximum score of 48.

High scores in the two subscales of emotional exhaustion and cynicism and low scores for professional efficacy permit us to lean towards a finding of burnout, although this has not been completely defined in the scientific literature. The instrument is considered valid with a Cronbach’s alpha of 0.86 for the dimension of emotional exhaustion, 0.76 for cynicism and 0.87 for professional efficacy [33].

The statistical treatment of MBI variables was performed with the average scores of each of the subscales. The scores of each dimension of the MBI were classified by terciles as low (lower tercile), medium (middle tercile) and high (upper tercile). For the analysis, people classified as the upper tercile of emotional exhaustion and cynicism and the lower tercile of professional efficacy have been grouped together as probable cases of burnout in order to associate this profile with the study variables.

Self-reported health (EQ-5D) [35] is a generic instrument for the measure of health-related quality of life that can be used both in individuals and in groups of patients, which comprises of two clearly distinct parts. In the first part of the instrument, respondents evaluate their health state, first in levels of severity by dimensions (descriptive system) with 5 items, each presenting 5 response options with 5 levels (level 1 indicates that there are no problems—level 5 indicates serious problems). Once the data have been gathered, the EQ index value is calculated for the state of health by the algorithm proposed by the EuroQol group, which reflects how good or bad a health state is according to the preferences of the general population of a country/region. The index oscillates between a value of 1 (best state of health) and 0 (death). Index values are a major feature of the EQ-5D instrument, facilitating the calculation of quality adjusted life years (QALYs) that are used to inform economic evaluations of healthcare interventions [36,37]. 

In a second section of the questionnaire, the EQ visual analogical scale (EQ VAS), the person must score his or her state of health on a scale from 0 (worst imaginable state of health) to 100 (best imaginable state of health) [36]. The EQ VAS provides a quantitative measure of the patient’s perception of their overall health [37]. The reliability coefficient of VAS calculated by Kappa coefficient is 0.82 (0.74–0.88 [37]).

### 2.4. Data Collection

Data were gathered by means of an ad hoc data collection notebook composed of a questionnaire that includes sociodemographic, occupational and health variables, along with validated questionnaires to measure dispositional optimism (LOT-R), the Maslach Burnout Inventory (MBI) and self-reported health (EQ-5D-5L). The research project was presented to the nursing management teams of long-term healthcare centers in Girona (Catalonia, Spain). All of these institutions agreed to participate in the research, and the administration and collection processes were carried out by the nursing directors of each of the centers.

The centers were first sent an electronic version of the data collection notebook to give them the opportunity to ask for clarification of any doubt as to how it should be filled in. The data collection notebook was understood and filled in without any changes being made to the items. A paper version was then sent to be distributed. Each envelope contained an information sheet for the participant, requesting their collaboration and the ad hoc data collection notebook. The answers were collected in well-identified locations in the different units during the three weeks post-delivery. A reminder was issued each week by email.

### 2.5. Ethics Statement

The study received approval from the ethics committee of the reference area (code number 138/12). The questionnaires were delivered individually in envelopes to nurses in their units and were collected 24 h later in the same units in closed envelopes. Spanish data protection legislation (LOPED 3/2018 of 5 December) was followed, guaranteeing the anonymity of the participants and of the centers as well as their right to withdraw from the study at any time. The research has applied the STROBE statement checklist for observational studies.

### 2.6. Data Analysis

Statistical analysis was performed with the SPSS Statistics® V19 software package (IBM Madrid, Spain). The Kolmogorov–Smirnov test was used to determine the normality of the distribution of the variables. Spearman’s rho test was used for the correlation of quantitative variables. Multiple linear regression models were used to determine health and burnout variables. In all cases, the level of significance (*p*) considered was < 0.05.

## 3. Results

In the present study, of the 156 nurses working in long-term healthcare centers in Girona, 69.87% responded (*n* = 109). Of the participants, 90.8% (*n* = 99) were women. The average age was 37.74 years (SD 10), and 50.45% of the nurses were between 36 and 66 years old. Of the nurses 73.39% perceived that they had a good or very good economic level, 59.63% had dependent family members and 18.3% reported having a chronic health problem. A total of 44.9% received continuous training, and 77.06% received social support whenever they needed it. A total of 60.55% did not have work-related family conflicts (Table 1).

The participants had an average score for optimism of 22.07 (SD 3.70). Nurses that did not have chronic health problems (*p* = 0.049), those that perceived having a good economic level (*p* = 0.003), and those that reported having social support whenever it was needed (*p* = 0.004) and did not present work-related family conflicts (*p* = 0.006) expressed having greater dispositional optimism (Table 1).

Participants that had chronic health problems (*p* = 0.013), those who received continuous training (*p* = 0.031) and those who reported having work-related family conflicts (*p* = 0.000) had the greatest emotional exhaustion. Nurses who were older (*p* = 0.022), had dependent family members (*p* = 0.012), received social support whenever they needed it (*p* = 0.042) and received ongoing training (*p* = 0.018) had greater professional efficacy (Table 1).

Better scores for self-reported health were obtained for younger nurses (*p* = 0.016), those that reported not to be suffering from chronic health problems (*p* < 0.001) and those that had more social support (*p* = 0.011). Scores for better perceived health (VAS) were higher for those that reported not having chronic health problems (*p* = 0.001), those that reported always having social support (*p* < 0.001) and those that reported not having work-related conflicts (*p* = 0.010; Table 2). 

Self-reported health (the EQ index value) correlated directly with dispositional optimism (Rho Spearman = 0.271; *p* < 0.01) and inversely with emotional exhaustion (Rho Spearman = −0.277; *p* < 0.01) and cynicism (Rho Spearman = −0.250; *p* < 0.01) (Table 3).

A total of 13.8% of probable cases of burnout were found (people with high levels of emotional exhaustion and cynicism and low levels of professional efficacy). Nurses with probable burnout had significantly lower dispositional optimism scores (*p* = 0.016). Furthermore, the index scores of self-reported health (*p* = 0.044) and the self-health assessment (*p* < 0.001) of the nurses who were not classified as suffering from burnout were higher than those of professionals classified as having burnout.

On performing the multiple linear regression model, it was found that the variables that are independently associated with better perceived health were social support, greater dispositional optimism and a lower level of emotional exhaustion or the absence of burnout (Table 4).

## 4. Discussion

In the study of dispositional optimism and burnout and its relationship with self-reported health, nurses with greater dispositional optimism have a greater perceived socioeconomic level and receive social support when they need it. In this respect, it is known that optimistic people have more and better social relationships [38]. It can be theorized that relationships with optimistic people are easier and that this fact has an influence over the greater social support they report [14]. The results also show that the most optimistic nurses do not report having work-related family conflicts. These results coincide with those reported by Carver et al. [14], who pointed to optimism as being a predictor of the resolution of family conflicts from any cause. If optimistic people satisfactorily resolve the conflicts that may arise in their lives, they are likely to have a lower perception of these conflicts. Subsequently, nurses who are less optimistic tend to see the negative aspects of an event and are more vulnerable to the stress of daily work. Therefore, being an optimist is an important characteristic that positively affects both personal and professional life.

Nurses reported low emotional exhaustion, and higher scores have been found in hospital nurses [39,40] and in mental health centers [40]. Participants with greater emotional exhaustion presented a higher perception of work-related family conflicts, a relationship that was also observed by Lee and Akhtar in hospital nurses [41]. These authors found an association between stressors in a social context and burnout in nurses, indicating that being affected in one’s personal life can cause burnout. In this respect, achieving a good balance between work and family life may reduce the emotional exhaustion of nurses. The levels of cynicism of the participants are considered to be average, as has also been described in hospital nurses [39]. High levels of cynicism have been described in nurses working in the field of mental health [40].

With regard to professional efficacy, the nurses in the study present high scores, as has also been described by several authors [39]. Lower scores were found in nurses who worked in mental health services [40]. While there is the possibility that cultural differences between organizations (for example, different leadership styles, rules and values) have a significant influence, the results may also suggest that nurses working in long-term healthcare feel more fulfilled in their work activity than nurses working in acute mental health centers.

The participants obtain higher scores for professional efficacy when they report receiving social support whenever they need it, a relationship that is supported by Garrosa et al. [42], who concluded that social support was a predictor of the professional efficacy of nurses. Nurses that perceived greater social support possibly perceive greater help in solving stressful situations, which in turn would have repercussions in helping them feel better at work.

In the present study, nurses with greater optimism and lower burnout presented greater self-reported health. Garrosa et al. [42] find optimism to be a factor that staves off emotional exhaustion among nurses and that actions should be considered that are aimed at increasing optimism in these professionals. Optimism has also recently been found to be an influential factor in nurses’ health [16]. In this respect, there are studies that suggest that interventions to increase optimism minimize the effects of burnout [8,43] although a strong relationship is not found between the two in the present study. Similarly, it appears that optimism in nurses is higher in nurses with better self-reported health [24]. It is important to note that the results of the multiple linear regression prove the association between optimism and the health of nurses. This result is in line with findings by other authors that optimism is associated with a greater perception of health in nurses with low levels of emotional exhaustion [44,45] and it seems to protect workers from the risk of job burnout [46]. Therefore, part of the loss of health of nurses working in the healthcare sector could be explained by the high emotional exhaustion that they present, as is supported by Khamisa et al. [23].

The prevalence of nurses with burnout is similar to that described in different international settings [23]. Although the relationship between emotional exhaustion and less social support in nurses has been reported [42], this was not found to be of statistical significance in our study. However, higher personal achievement scores are obtained when nurses report receiving social support whenever they need it, such as in Garrosa et al. [42], who concluded that social support was a predictor of the personal fulfillment of nurses.

These results are important as very few studies in the area of geriatrics have studied the relationship between burnout, perceived health and optimism [47,48]. With regards to Spain itself, where this study has been conducted, no previous studies have studied dispositional optimism, burnout and perceived health in geriatric care. Our group had analyzed the relationship between these constructs but optimism was studied only through a single question and in the area of hospital-based nursing care [7].

### 4.1. Limitations of the Study

The limitations of the present study are methodological and are related to the design and size of the sample. The cross-sectional design has allowed an approximation to the reality of nurses with regards to dispositional optimism, burnout and their association with health that opens up future lines of research in which longitudinal studies will make it possible to establish cause–effect relationships between the variables.

With regards to the sample size, the number of participants could limit the results of the study, although it should be taken into account that the response rate is high with regards to the total population of the studied area.

Finally, the lack of consensus regarding the interpretation of some of the instruments used, such as the LOT-R, makes it difficult to make comparisons with other published studies. 

### 4.2. Practical Implications

Nurse managers in the healthcare sector should plan specific interventions to increase optimism, such as cognitive activities that generate positive emotions [10]. Study results suggest that interventions planned from leadership positions for the increase of optimism among other positive variables are effective in minimizing the effects of burnout [43], thus contributing to the improvement of the occupational health of nurses and to the promotion of a healthy work environment.

With regards to burnout, the levels that are found suggest that this syndrome is not widely recognized in the working environments of these nurses and that measures should be taken so that nurses can be treated at an early stage both for their own benefit and to maintain the quality of care of the people under their charge.

## 5. Conclusions

The present study is the first to study dispositional optimism and burnout and their relationship with the health of long-term healthcare nurses in Spain. Nurses with greater dispositional optimism have a higher perceived socioeconomic level, report receiving social support and do not manifest work-related family conflicts. They also present greater professional efficacy at work and self-reported health and so optimism is revealed as an interesting variable modulating the occupational health of nurses. The results obtained should act as a stimulus to promote interventions aimed at motivating nurses.

## Figures and Tables

**Table 1:** Dispositional optimism and burnout scores by sociodemographic, occupational and health variables (*n* = 109).

Variables	Total Population (%)	Dispositional Optimism	*p*	Emotional Exhaustion	*p*	Cynicism	*p*	Professional Efficacy	*p*
**Sex**									
Men	10 (9.17)	22.70 (4.02)	0.860	13.00 (10.22)	0.672	7.20 (5.82)	0.426	41.80 (6.42)	0.860
Women	99 (90.82)	22.01 (3.69)		14,54 (10,95)		5.97 (4.52)		40.61 (5.89)	
**Age**									
21 to 35 years	54 (49.54)	22.33 (3.82)	0.888	14.33 (11.10)	0.954	6.39 (4.25)	0.497	39.41 (5.99)	0.022 *
36 to 66 years	55 (50,45)	21,82 (3.60)		14,45 (10,65)		5.78 (5.00)		42,00 (5,62)	
**Socioeconomic level**									
Very good or good	80 (73.39)	22.70 (3.45)	0.003 *	13.49 (10.27)	0.148	5.99 (4.66)	0.724	40.83 (5.82)	0.751
Regular or bad	29 (26.61)	20.34 (3.88)		16.90 (12.13)		6.34 (4.63)		40.41 (6.30)	
**Civil status**									
Married	51 (46.78)	21.76 (3.57)	0.800	15.80 (10.88)	0.087	6.16 (4.77)	0.481	40.94 (5.70)	0.824
Single	44 (40.36)	22.39 (4.07)		14,27 (11,24)		6.43 (4.82)		40,30 (6,17)	
Other	14 (12.85)	22.21 (3.06)		9.64 (8.48)		4.71 (3.40)		41.21 (6.30)	
**Dependent family members**									
Yes	65 (59.63)	22.09 (3.61)	0.925	14.94 (10.93)	0.309	5,88 (4,76)	0.626	41,92 (5,28)	0.012 *
No	44 (40.37)	22,02 (3.93)		12.84 (9.69)		6.33 (4.51)		39 (6.47)	
**Chronic health problem**									
Yes	20 (18.34)	20.60 (3.26)	0.049 *	19.80 (12.10)	0.013 *	6.70 (5.94)	0.596	39.95 (5.64)	0.525
No	89 (81.66)	22.40 (3.73)		13.18 (10.23)		5.94 (4.31)		40.89 (6)	
**Social support**									
Yes, always	84 (77.06)	22.67 (3.49)	0.004 *	13.26 (10.86)	0.072	5.89 (4.50)	0.573	41.37 (5.51)	0.042*
Sometimes /Never	24 (22.93)	20.25 (3.76)		17.54 (9.76)		6.50 (5.08)		38.58 (6.94)	
**Studies**									
Diploma or degree	98 (89.90)	21.99 (3.63)	0.737	14.12 (10.36)	0.437	6.11 (4.66)	0.843	40.55 (5.97)	0.389
Master’s degree	11 (10.01)	22.82 (4.46)		16.82 (14.91)		5.82 (4.60)		42.18 (5.51)	
**Years in the profession**									
0 to 10 years	42 (38.53)	22.17 (3.87)	0.749	11.68 (9.48)	0.055	6.12 (4.70)	0.949	39.98 (6.14)	0.305
More than 10 years	67 (61.46)	22.01 (3.62)		15.97 (11.41)		6.06 (4.63)		41.18 (5.78)	
**Contractual status**									
Permanent position	86 (78.89)	21.69 (3.54)	0.431	14.89 (10.56)	0.395	6.00 (4.69)	0.741	40.98 (6.12)	0.415
Eventual	23 (21.10)	23.31 (4.01)		12.81 (11.80)		6.35 (4.54)		39.88 (5.27)	
**Category within the team**									
One single function	77 (70.64)	22.49 (3.43)	0.059	13.12 (9.62)	0.056	6.52 (4.85)	0.128	40.55 (6.06)	0.644
More than one function	32 (29.36)	21.06 (4.18)		17.47 (13.00)		5.03 (3.94)		41.13 (5.65)	
**Continuous training**									
Yes	49 (44.95)	21.92 (3.82)	0.529	12.18 (10.46)	0.031 *	5.51 (4.32)	0.267	41.96 (5.83)	0.018 *
No	50 (55.05)	22.38 (3.44)		16.80 (10.50)		6.52 (4.66)		39.08 (6.11)	
**Work-related family conflicts**									
Yes	66 (60.55)	21.29 (3.67)	0.006 *	17.44(11.36)	<0.001 **	6.61 (4.95)	0.145	40.41 (5.73)	0.506
No	43 (39.45)	23.28 (3.46)		9.72 (8.12)		5.28 (4.02)		41.19 (6.25)	

Student’s *t*-test. Quantitative variables are expressed as mean and SD in brackets. * Statistical significance *p* < 0.05 and ** statistical significance *p* < 0.001.

**Table 2:** Self-reported health scores by sociodemographic, occupational and health variables (*n* = 109).

Variables	Index Self-Reported Health	*p*	VAS	*p*
**Sex**				
Men	0.949 (0.057)	0.326	85.32 (11.66)	0.532
Women	0.915 (0.109)		87.70 (8.20)	
**Age**				
21 to 35 years	0.942 (0.087)	0.016 *	87.30 (10.30)	0.111
36 to 66 years	0.894 (0.177)		83.32 (12.17)	
**Socioeconomic level**				
Very good or good	0.952 (0.090)	0.066	85.49 (11,22)	0.935
Regular or bad	0.887 (0.139)		85.69 (12)	
**Civil status**				
Married	0.944 (0.075)	0.092	87.32 (11.77)	0.118
Single	0.904 (0.105)		83.18 (11.36)	
Other	0.885 (0.164)		88.57 (9.07)	
**Dependent family members**				
Yes	0.907 (0.116)	0.153	84.26 (11.08)	0.109
No	0.937 (0.870)		87.84 (11.48)	
**Chronic health problems**				
Yes	0.839 (0.122)	<0.001 **	79.00 (10.07)	0.004 *
No	0.936 (0.093)		87.01 (11.18)	
**Social support**				
Yes, always	0.931 (0.104)	0.011 *	87,67 (8,74)	0.000 **
Sometimes	0.870 (0.100)		77.92 (15.94)	
**Studies**				
Diploma or degree	0.916 (0.108)	0.565	85.30 (11.69)	0.504
Master’s degree	0.931 (0.089)		87.73 (8.17)	
**Years in the profession**				
0 to 10 years	0.946 (0.728)	0.025 *	85.10 (13.78)	0.748
More than 10 years	0.900 (0.119)		85.82 (9.67)	
**Contractual status**				
Permanent position	0.913 (0.108)	0.424	85.06 (11.41)	0.433
Eventual	0.932 (0.097)		87.08 (11.37)	
**Category within the team**				
One single function	0.928 (0.094)	0.123	86.44 (10.39)	0.202
More than one function	0.893 (0.123)		83.38 (13.39)	
**Receiving continuous training**				
Yes	0.911 (0.098)	0.629	85.45 (11.25)	0.892
No	0.921 (0.112)		85.14 (11.25)	
**Work-related family conflicts**				
Yes	0.911 (0.098)	0.416	83.29 (11.35)	0.010 *
No	0.928 (0.117)		89 (10.63)	

Student’s *t*-test. Quantitative variables are expressed as mean and SD in brackets. * Statistical significance *p* < 0.05 and ** statistical significance *p* < 0.001.

**Table 3:** Dispositional optimism, burnout and self-reported health (*n* = 109).

Variables	Mean	SD	1	2	3	4	5	6
1. Self-reported health	0.918	0.106	1	0.549 **	0.271 **	−0.277 **	−0.250 **	0.158
2. EQ VAS	85.54	11.38		1	0.318 **	−0.451 **	−0.175	0.189 *
3. Dispositional optimism	22.07	3.70			1	−0.352 **	−0.183	0.237 *
4. Emotional exhaustion	14.39	10.85				1	0.537 **	−0.449 **
5. Cynicism	6.08	4.63					1	−0.415 **
6. Professional efficacy	40.72	2.40						1

Rho Spearman correlation: SD: standard deviation; VAS: Visual analogue scale for EQ-5D-5L. * Statistical significance *p* < 0.05 and ** statistical significance *p* < 0.001.

**Table 4:** Lineal regression models for self-reported health, dispositional optimism and burnout (*n* = 109).

Variables	Dependent Variable: Self-Reported Health (EQ VAS)							
	B	EE	IC 95%	*p*	B	EE	IC 95%	*p*
Age	−0.07	0.1	0.27–0.13	0.501	−0.07	0.1	−0.27–−0.12	0.478
Sex	1.33	3.42	−5.46–8.12	0.698	1.11	3.35	−5.53–7.77	0.739
Social support	−4.44	2.39	−9.19–−0.29	0.066	−4.78	2.32	−9.38–0.18	0.042
Dispositional Optimism	0.66	0.29	0.83–1.24	0.026	0.68	0.28	0.12–1.24	0.018
Emotional Exhaustion	−3.33	0.29	−5.90–0.76	0.011				
Burnout					−10.6	3.19	−16.94–−4.26	0.001
R^2^		0.227				0.256		
Corrected R^2^		0.189				0.220		

B: coefficient B; EE: standard error; CI 95%: confidence interval of 95%.VAS: visual analogue scale for EQ-5D-5L. R^2^: R-Squared, the coefficient of determination; corrected R^2^: adjusted R-Squared (the adjusted coefficient of determination).
